# Development and Validation of HPLC-DAD and UHPLC-DAD Methods for the Simultaneous Determination of Guanylhydrazone Derivatives Employing a Factorial Design

**DOI:** 10.3390/molecules22091394

**Published:** 2017-08-30

**Authors:** Wanessa Azevedo de Brito, Monique Gomes Dantas, Fernando Henrique Andrade Nogueira, Edeildo Ferreira da Silva-Júnior, João Xavier de Araújo-Júnior, Thiago Mendonça de Aquino, Êurica Adélia Nogueira Ribeiro, Lilian Grace da Silva Solon, Cícero Flávio Soares Aragão, Ana Paula Barreto Gomes

**Affiliations:** 1 Drug Quality Control Laboratory (LCQMed), Pharmaceutical Sciences Department, Federal University of Rio Grande do Norte–UFRN, Av. General Cordeiro de Faria s/n, Natal RN 59012-570, Brazil; wanessabrito@pmenos.com.br (W.A.d.B.); monique_dantasrn@hotmail.com (M.G.D.); fhanogueira@gmail.com (F.H.A.N.); cicero.aragao@yahoo.com.br (C.F.S.A.); 2 Laboratory of Medicinal Chemistry, Nursing and Pharmacy School, Federal University of Alagoas, ESENFAR, Av. Lourival de Mello Motta s/n, Maceió AL 57072-970, Brazil; edeildojr@hotmail.com (E.F.d.S.-J.); jotaaraujo2004@gmail.com (J.X.d.A.-J.); thiago.aquino@iqb.ufal.br (T.M.d.A.); euricanogueira@gmail.com (Ê.A.N.R.); 3 Pharmaceutical Sciences Department, Federal University of Amapá–UNIFAP, Rod Juscelino Kubitschek, Km-02, Macapá AP 68903-419, Brazil; liliansolon@yahoo.com.br

**Keywords:** guanylhydrazones, factorial design, HPLC-DAD, UHPLC-DAD, method validation

## Abstract

Guanylhydrazones are molecules with great pharmacological potential in various therapeutic areas, including antitumoral activity. Factorial design is an excellent tool in the optimization of a chromatographic method, because it is possible quickly change factors such as temperature, mobile phase composition, mobile phase pH, column length, among others to establish the optimal conditions of analysis. The aim of the present work was to develop and validate a HPLC and UHPLC methods for the simultaneous determination of guanylhydrazones with anticancer activity employing experimental design. Precise, exact, linear and robust HPLC and UHPLC methods were developed and validated for the simultaneous quantification of the guanylhydrazones LQM10, LQM14, and LQM17. The UHPLC method was more economic, with a four times less solvent consumption, and 20 times less injection volume, what allowed better column performance. Comparing the empirical approach employed in the HPLC method development to the DoE approach employed in the UHPLC method development, we can conclude that the factorial design made the method development faster, more practical and rational. This resulted in methods that can be employed in the analysis, evaluation and quality control of these new synthetic guanylhydrazones.

## 1. Introduction

**Figure 1:**
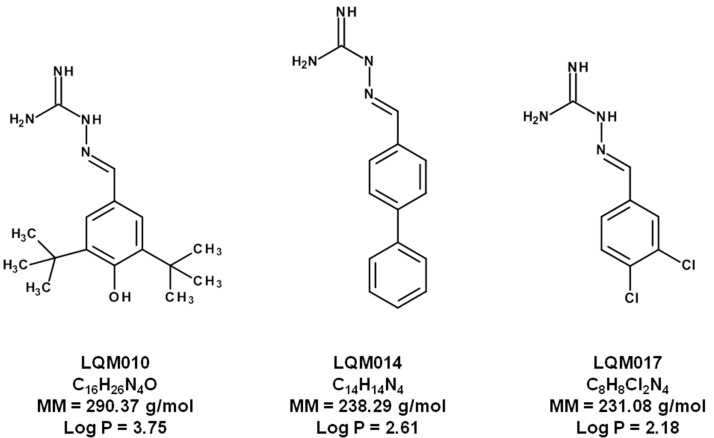
Chemical structures, molecular masses (MM) and partition coefficients (log*P*) of the guanylhydrazones LQM010, LQM014 and LQM017.

Guanylhydrazones are widely studied molecules of great scientific interest due to their broad biological potential, which includes antihypertensive, antidiabetic, antimalarial, trypanocidal, anti-bacterial and antitumoral activities [1,2,3,4,5,6,7,8,9,10]. Andreani et al. synthesized and evaluated the biological activity of some new guanylhydrazones against a series of lung, breast and glioma tumors. They concluded that the 3-nitrophenyl and 4-nitrophenyl moieties are important pharmacophore groups which increase the affinity for specific tumor receptors [11]. Other research groups have synthesized new guanylhydrazones with anticancer activity in order to obtain new molecules for the treatment of this disease [12]. The guanylhydrazone derivatives 2-[(3,5-di-*tert*-butyl-4-hydroxyphenyl)methylene]hydrazinecarboximidamide (LQM10), 2-([1,10-biphenyl]4-ylmethylene)hydrazinecarboximidamide (LQM14) and 2-[(3,4-dichlorophenyl)-methylene]hydrazinecarboximidamide (LQM17) have shown pharmacological activity against some neoplastic cell lines, such as human colon (HCT-8), melanoma (MDA-MB435), glioblastoma (SF-295) and promyelocytic leukemia (HL-60) [12]. The chemical structures of LQM10, LQM14 and LQM17 are shown in Figure 1, along with their molecular masses and partition coefficients (log*P*).

The quality of pharmaceutical raw materials is a constant concern mainly in the process of drug synthesis, in which the synthetic route can lead to the formation of chemically-related substances and impurities. It has been shown that in the synthetic process, some guanylhydrazones may be generated and become impurities of others. Galvão et al evaluated some guanylhydrazones employing thermal analysis and observed that some guanylhydrazones other than the main product were present in very small quantities. These data were confirmed by HPLC analysis [13].

Thus, the use of validated analytical methods to evaluate the quality of pharmaceutical raw materials is a necessary condition in the process of obtaining an adequate product. Amongst the various analytical techniques available, high performance liquid chromatography (HPLC) stands out as an excellent, precise and robust separation technique, with excellent applicability for raw material quantification (quality control), in process control and quality control of pharmaceutical products [14,15,16,17,18]. The desire to obtain information more quickly and with less solvent consumption has led to advances in chromatography, which resulted in the development of ultra-high performance chromatography (UHPLC). UHPLC is based on the use of pumps that can operate at higher pressures and columns with smaller particles, with diameters lower than 2.2 µm, allowing higher efficiency and resolution and shorter run times [19]. There are some analytical methods for the quantification of commercially available synthetic guanylhydrazones by HPLC [9,20,21], although there are few methods for the quantification of the recently synthesized ones, but there are no analytical methods for the analysis of the guanylhydrazones reported in this work.

As the current concern is to obtain clean techniques and use less solvent, the factorial design of experiments has become an essential tool in the optimization of methods to meet the demands of green chemistry. In a factorial design of experiment several factors can be evaluated simultaneously to obtain a response. In a chromatographic method, the factors that must be determined to establish the optimal conditions of analysis are, among others, temperature, mobile phase composition, mobile phase pH, and column length [17]. The main advantage of employing a factorial design of experiments over the one factor at a time approach is that the influence of one factor into another can be calculated. Besides that, the number of experiments is limited [22]. Two uses of DoE in chromatography are for demonstrating a lack of significant effects in robustness studies for method validation, and for identifying significant factors and then optimizing a response with respect to them during the method development [23].

The aim of the present work was to develop and validate a HPLC and an UHPLC method for the simultaneous determination of guanylhydrazones with anticancer activity employing an experimental design. The HPLC method was developed empirically, while design of experiments (DoE) was employed for the UHPLC method development.

## 2. Results

### 2.1. Development and Validation of the HPLC Method

#### 2.1.1. Optimization of the Chromatographic Conditions

**Figure 2:**
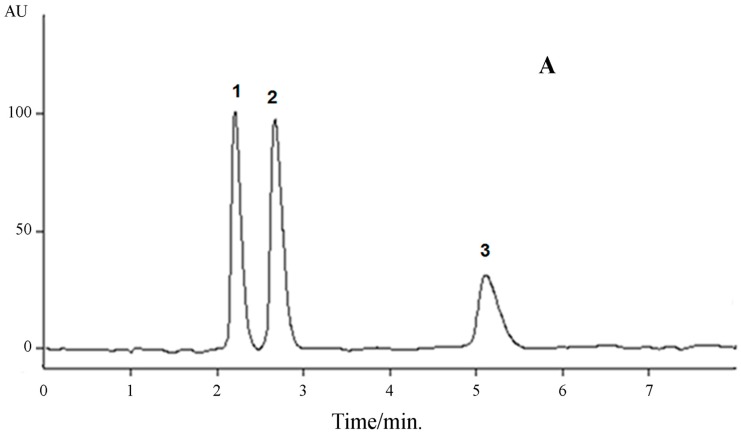

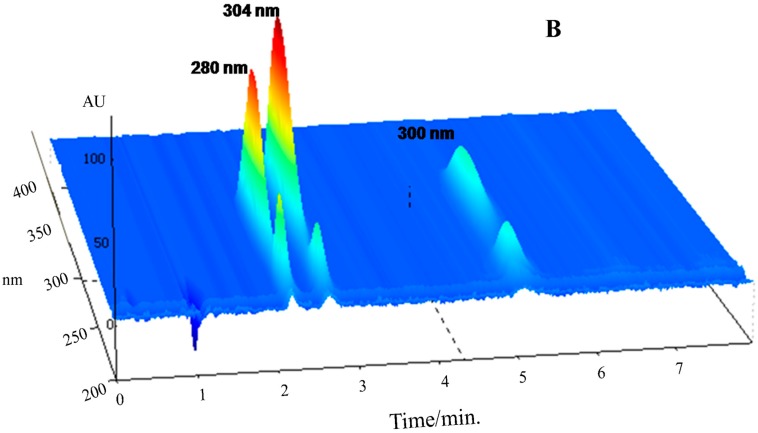
Chromatogram (2D—(**A**) and 3D—(**B**)) of the separation of the guanylhydrazones by HPLC/DAD.

The mobile phase was chosen after several trials with acetonitrile, methanol and water in various proportions. In addition, different types of acid modifiers were added to adjust the pH of the mobile phase. A mobile phase consisting of pH 3.5 (adjusted with acetic acid) methanol−water (60:40 *v*/*v*) at ambient temperature was finally selected, which produced optimal separation, high sensitivity and good peak shape. The addition of acetic acid was indispensable to allow suitable peak symmetry and resolution. The bi-dimensional UV absorption spectra of the studied compounds showed that 290 nm was the wavelength of maximum absorbance for the quantification of all compounds of interest. A representative chromatogram of the separation of the guanylhydrazones by HPLC is shown in Figure 2.

#### 2.1.2. HPLC Method Validation

##### Selectivity

Satisfactory results were obtained, indicating the high selectivity of the proposed method for the simultaneous determination of LQM10, LQM14 and LQM17 (Table 1). The retention times were 2.18, 2.64 and 5.08 minutes for LQM17, LQM14 and LQM10, respectively. No interference between the guanylhydrazones was found, showing that the peaks were free from coelution. The similarity indexes for LQM10, LQM14 and LQM17 were 959, 973 and 979, respectively, indicating that none of the guanylhydrazones studied coeluted.

##### Linearity

The curves were linear within the concentration range studied (1–25 µg mL^−1^) for each analyte and high values of regression coefficient were obtained, superior to 0.999 for each guanylhydrazone studied (Table 1).

##### LOD and LOQ

The limits of detection for the guanylhydrazones LQM10, LQM14, and LQM17 were 0.15, 0.08 and 0.12 µg·mL^−1^, respectively. The limits of quantification for the guanylhydrazones LQM10, LQM14, and LQM17 were 0.51, 0.27 and 0.39 µg·mL^−1^, respectively.

##### Precision

RSD’s for intra-day precision for the three guanylhydrazones ranged from 1.24 to 2.00% and those for the inter-day precision ranged from 1.56 to 2.81%. These results indicated the high precision of the method.

##### Accuracy

Accuracy was determined employing a standard addition experiment. The data on accuracy is shown in Table 1 confirming that all the recovery percentages were within the acceptable range. Therefore, the method was accurate with recovery rates from 98.69% to 101.47% for samples spiked with guanylhydrazones.

##### Robustness

Robustness was determined by analyzing the same samples under the deliberate variation of method conditions and parameters. These variations did not have any significant effect on the measured responses nor on the chromatographic resolution. RSD% for the measured peak areas did not exceed 2.54% (Table 1).

### 2.2. Experimental Design and Validation of UHPLC Method

#### 2.2.1. Response Surface Methodology (RSM) and UHPLC Method Development

**Figure 3:**
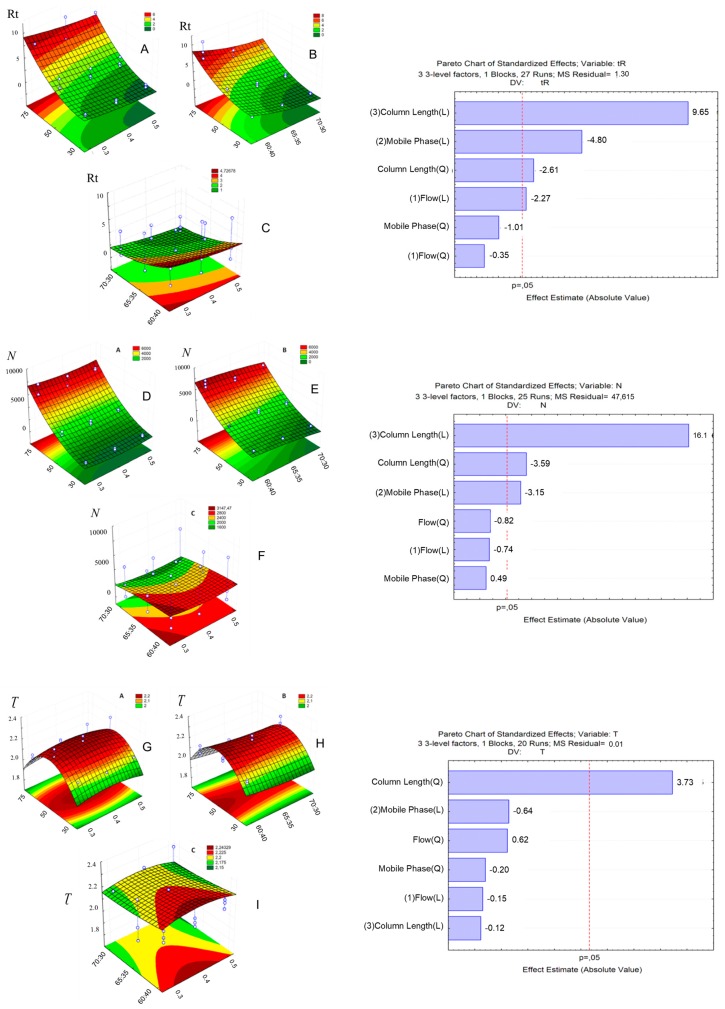
Factorial experimental design to UHPLC method development. The effects Rt (**A**)–(**C**), N (**D**)–(**F**), Ʈ (**G**)–(**I**) and response surface and Pareto graphs.

A factorial experimental design (Figure 3) was employed in the UHPLC method development to reduce the number of experiments and to evaluate the effects of the following parameters: column length, mobile phase flow rate and mobile phase composition.

**Figure 4:**
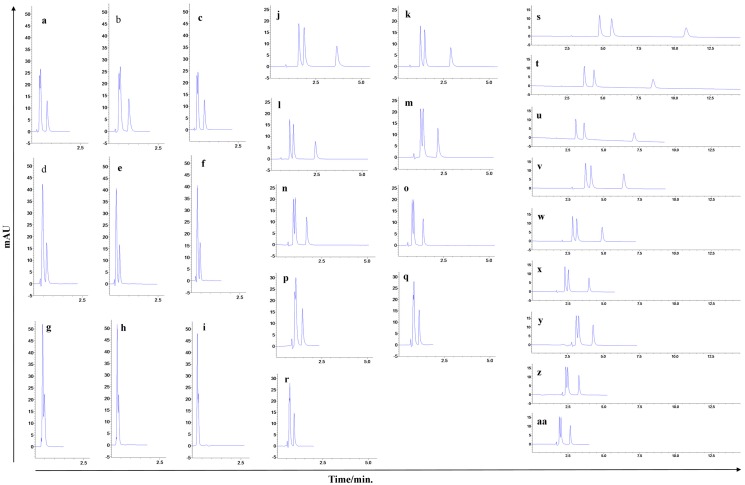
Chromatograms obtained by conditions design of experiments (DoE) approach to development of an UHPLC method with the 30 mm (**a**–**i**), 50 mm (**j**–**r**) and 75 mm (**s**–**aa**) column length. Chromatograms (**a**–**i**), (**j**–**r**) and (**s**–**aa**) are respectively in the same scale.

Peak coelution was observed in the chromatograms obtained with a 30 mm column (Figure 4a–i). In this column, the separation efficiency of the compounds was greatly compromised, with problems in peak resolution independently of the chromatographic condition employed, resulting in coelution of the first two peaks in most of the runs and coelution of the three peaks when the organic solvent proportion was at its maximum (70% MeOH). Figure 3 shows the effects of column length, mobile phase flow rate and mobile phase composition on retention time. The effects were evaluated in the response surface and Pareto graphs.

We verified that the increase in column length was directly proportional to the increase in retention time, due to an increase in the theoretical plate number, independently of the mobile phase flow rate employed (Figure 3A,D). However, a reduction in the organic solvent proportion of the mobile phase produces a small reduction in the retention time in relation to the column length (Figure 3B). These results confirmed that the column length is decisive in the separation efficiency and analytes’ retention time, because it is known that longer columns present a greater theoretical plate number, producing better resolution values although resulting in longer run times [24].

The chromatograms obtained with the 50 mm column presented shorter retention times, although the complete separation of the three peaks was only possible with a lower proportion of the organic solvent (60% MeOH), independently of the mobile phase flow rate employed (Figure 4j–r). The greater the organic solvent proportion in mobile phase, the shorter the analyte interaction with the stationary phase and the shorter is the separation efficiency, despite of a shorter run time.

**Figure 5:**
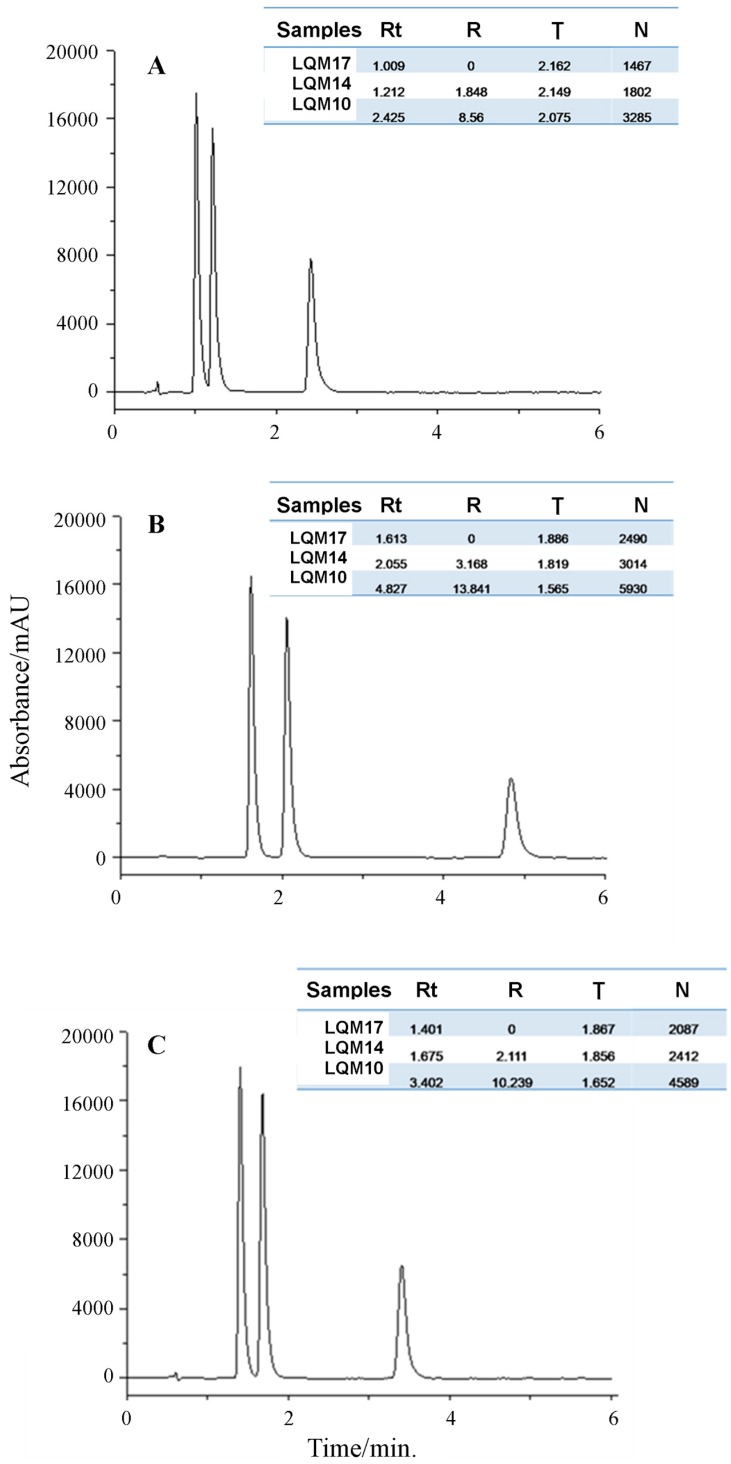
Chromatograms of the best UHPLC method obtained under different conditions to mobile phase composition: (**A**) MeOH:H_2_O (60:40) pH 3.5; (**B**) MeOH:H_2_O (60:40) + 0.1% TEA and (**C**) MeOH:H_2_O (65:35) + 0.1% TEA. Column length: 50 mm; the mobile phase flow rate: 0.5 mL·min^−1^; UV detection wavelength: 290 nm.

The best chromatogram obtained was the one that employed a 50 mm column, mobile phase proportion of 60:40, flow rate of 0.5 mL·min^−1^, although the peaks were tailed, with tailing factor of more than 2.0. The excessive peak tail compromised the resolution between the first two peaks (R < 2.0) what compromised also the peak integration and, consequently, the system reproducibility [25]. Besides that, the theoretical plate number was inadequate for LQM17 and LQM14. Tailed peaks are generally caused by the analytes’ secondary interactions with the stationary phase. It’s called chemical tail when a basic analyte, positively charged, interacts with the residual silanols of the stationary phase. It’s recommended to increase the mobile phase pH, add an amine modifier such as triethylamine 0.1% or to employ a column with less silanophilic activity to reduce peak tail [24,26]. Thus, triethylamine was added to the aqueous phase of mobile phase to act as a competitive base and to reduce the column silanol group exposure (Figure 5).

The TEA addition solved the problem of peak tailing and increased the peak´s theroretical plate number; however, the run time was also increased. The increase of run time was an undesirable consequence of TEA addition to the mobile phase. Thus, the eluotropic strength was increased to the intermediate level of the experimental design (65:35) and the flow rate was maintained at 0.5 mL·min^−1^ to reduce the run time without compromising the separation. It was possible to achieve a chromatogram (Figure 5C) with good peak resolution, short run time and an adequate tailing factor employing these conditions, which allowed the method to be validated.

#### 2.2.2. UHPLC Method Validation

##### Selectivity

The UHPLC method for guanylhydrazones was also selective. The similarity index for LQM10, LQM14 and LQM17 were 999, 999 and 1000, respectively, indicating that the studied guanyl- hydrazones did not coelute (Table 1). The retention times were 1.40, 1.67 and 3.40 minutes for LQM17, 14 and 10, respectively.

##### Linearity

The curves were linear within the concentration range studied for each analyte (1–25 µg mL^−1^) and high values of regression coefficient were obtained, superior to 0.999 for each guanylhydrazone studied (Table 1).

##### LOD and LOQ

The limits of detection for the guanylhydrazones LQM10, LQM14 and LQM17 were 0.02, 0.04 and 0.08 µg·mL^−1^. The limits of quantification for the guanylhydrazones LQM10, LQM14 and LQM17 were 0.06, 0.15 and 0.28 µg·mL^−1^ (Table 1).

##### Precision

RSD’s for intra-day precision for the three guanylhydrazones determination ranged from 0.53 to 1.27% and those for the inter-day precision ranged from 0.43 to 0.68%. These results indicated a better precision of the UHPLC method than the HPLC method (Table 1).

##### Accuracy

The method was accurate with recovery rates from 99.32% to 101.62% for samples spiked with guanylhydrazones (Table 1).

##### Robustness

Deliberate variations on the UHPLC chromatographic conditions (pH and flow rate) did not produce any significant effect on the measured responses nor on the peak resolution. RSD% for the measured peak areas did not exceed 1.93% (Table 1). Some considerations can be made after the HPLC and UHPLC methods validation. One of them is the run time reduction in the UHPLC method, what lead to a 3.7 times reduction in solvent consumption. The lower flow rate employed also contributed to this reduction. This finding is relevant, since solvent disposal is a worldwide concern. It was also possible to reduce the injection volume in UHPLC, and it was only possible because the UHPLC apparatus is equipped with an auto injector, which is able to inject minimal sample volumes while the HPLC apparatus is equipped with manual injector. The reduction of the injection volume in UHPLC contributed to enhance the separation efficiency.

## 3. Material and Methods

### 3.1. Chemicals

HPLC grade Acetonitrile from J.T. Baker (Phillipsburg, NJ, USA), trifluoroacetic acid (Halocarbon, North Augusta, SC, USA) and hydrochloric acid from J.T. Baker (Phillipsburg, NJ, USA) were used. Ultrapure water was obtained from a Milli-Q Integral Water Purification System (Millipore, Bedford, MA, USA). All guanylhydrazones were synthesized as described in the literature [6] and their purity was confirmed by elemental analysis, ^1^H-NMR and melting point. All other reagents were of analytical grade.

### 3.2. Instrumental and Analytical Conditions

HPLC Analyses were carried out on a SYKAM HPLC System (Eresing, Germany) composed of a S7131 pump, a Rheodyne manual injector and a S3240 photodiode-array detector (DAD). UV spectra from 200 to 400 nm were online recorded for peak identification, for peak purity calculation and to select the wavelength that provided the best sensitivity for all the investigated compounds. Chromatographic separations were achieved on a C18 ACE analytical column (150 mm × 4.6 mm i.d. 5 μm particle size). Equipment control, data acquisition and integration were performed with Clarity software (DataApex, Prague, Czech Republic). UHPLC analyses were carried out on a Shimadzu UFLC-XR^®^ system (Shimadzu, Kyoto, Japan), equipped with two LC 20-ADXR solvent delivery units, autosampler (SIL-20ACXR), degassing unit (DGU-20A3), column oven (CTO-20 AC) and photodiode-array detection (SPD-M20A). Three columns were employed in the method development (Shim-pack XR-ODS, 2.2 µm particle size) with different dimensions: 30 mm × 2.0 mm i.d., 50 mm × 3.0 mm i.d. and 75 mm × 4.6 mm i.d. Samples and solvents were filtered in 0.22 µm syringe filters (Millipore) and degassed in sonic bath (QUIMIS-Q3350).

### 3.3. Preparation of Sample Solution

The stock solution was prepared by weighing accurately, 5.0 mg of each guanylhydrazone and transferring it to a 10 mL volumetric flask. After the addition of 5 mL of mobile phase, the flasks were sonicated for 15 min. The samples were made up to volume with mobile phase. The stock solution was then diluted to the work concentration (10 µg·mL^−1^) with mobile phase.

### 3.4. Methods Development

#### 3.4.1. HPLC Method Development

The ideal HPLC conditions were selected arbitrarily to allow the complete separation of the three analytes. Two stationary phases (C8 and C18) and two mobile phases (ACN:H_2_O and MeOH:H_2_O) were tested. At the same time, the mobile phase flow rate (from 0.8 to 1.8 mL min^−1^) and pH (5.0 and 3.5) were varied in order to select the optimal values. The guanylhydrazone samples were initially injected individually, to observe their retention times and the UV spectrum obtained from the DAD. These results allowed us to establish the samples elution order, making their identification in the mixture easy.

#### 3.4.2. UHPLC Method Development

The HPLC conditions achieved initially were the basis for the development of an UHPLC method, employing a design of experiments (DoE) approach. A three factor (column length, flow rate and mobile phase composition) experimental design was performed in three levels, as shown in Table 2. The sample volume (1.0 µL), the work concentration (10 µg mL^−1^), mobile phase pH (3.5, adjusted with acetic acid) and column temperature (25 °C) remained constant during all the chromatographic runs. The response variables were the retention time (Rt), theoretical plate number (N), tailing factor (Ʈ) and the critical peak pair resolution (R). The 3^3^ experimental design resulted in 27 chromatographic runs in order to evaluate the influence of each factor in the guanylhydrazones chromatographic behavior. The results contributed to the development of the method. The method was then validated.

### 3.5 Method Validation

Validation was performed according to the ICH guidelines [15]. The validation parameters evaluated were specificity, linearity, accuracy, precision (intra and inter-day) and robustness.

#### 3.5.1. Specificity/Selectivity

The specificity and selectivity of the method were evaluated by comparing the chromatograms of the three isolated guanylhydrazones with the chromatogram of a sample containing the three guanylhydrazones mixed. The aim of this experiment was to evaluate the absence of any interference in each analyte peaks. Spectral scans were collected over the range of 200–400 nm by the diode array detector operating in full-scan mode. The UV spectrum of each guanylhydrazone was recorded and the spectral purity (similarity index) was calculated.

#### 3.5.2. Linearity

The linearity of the method was confirmed using standard solutions at seven different concentrations of guanylhydrazones within the range of 1–25 µg·mL^−1^ (1, 2, 5, 10, 15, 20 and 25 µg·mL^−1^). Each solution was analyzed in triplicate. The assays were performed according to previously established experimental conditions. Mean peak area values were plotted against the corresponding analyte concentration. The obtained data were subjected to regression analysis using the least-squares method and the coefficient of determination (*r^2^*) for each guanylhydrazone was evaluated.

#### 3.5.3. Limit of Detection (LOD) Limit of Quantification (LOQ)

The limit of detection (LOD) and the limit of quantification (LOQ) are considered the lowest compound concentration detectable and the exact value determinable with suitable precision and accuracy, respectively. The LOD and LOQ were determined as the lowest concentrations of the analyte that provided signal-to-noise ratios of 3:1 and 10:1, respectively [25].

#### 3.5.4. Precision

The precision of an analytical procedure expresses the closeness of agreement between a series of measurements obtained from multiple sampling of the same homogeneous sample under the prescribed conditions. According to the ICH, the precision of the developed method was estimated by calculating repeatability (intra-day precision) and the intermediate precision (inter-day precision). The intra-day precision was determined as the RSD calculated from the analyses of six individually prepared standard solutions of the three guanylhydrazones at the work concentration (10 µg mL^−1^) in the same day. The same experiment was repeated the next day by another analyst to determine inter-day precision.

#### 3.5.5. Accuracy

Accuracy is assessed by the proximity of the obtained value to the ‘true’ value and can be reported in terms of recovery by adding known amounts of the studied compounds to a known concentration of the substances (standard addition method). The resulting mixtures were assayed and the mean recoveries and their standard deviation for three replicates were calculated by the relationship between the experimental concentration and the theoretical concentration expressed as a percentage ((*C*_experimental_/*C*_theoretical_) × 100) after which it was analyzed, along with its relative standard deviations.

#### 3.5.6. Robustness

The robustness of an analytical procedure is a measure of its capacity to remain unaffected by small, but deliberate variations in method parameters and provides an indication of its reliability during normal usage [25]. Therefore, the guanylhydrazones solutions (n = 6) were prepared and analyzed employing both the established conditions and under variation of mobile phase pH and flow rate. By HPLC the mobile phase pH was modified to 3.45 and 3.55; the flow rate of the mobile phase employed was 1.45 and 1.55 mL min^−1^ for robustness evaluation. By UHPLC the mobile phase pH was modified to 3.45 and 3.55; the flow rate of the mobile phase employed was 0.45 and 0.55 mL min^−1^ for robustness evaluation.

## 4. Conclusions

Precise, exact, linear and robust HPLC and UHPLC methods were developed and validated for the simultaneous quantification of the guanylhydrazones LQM10, LQM14, and LQM17. The UHPLC method is more economic, with four times less solvent consumption, and with a 20 times less injection volume, which allowed a better column performance. The developed methods can be considered cost and time effective, because they are isocratic and have a simple mobile phase composition, the samples are prepared without the need of various steps and great time of analysis. This resulted in methods that can be employed in the analysis, evaluation and quality control of these new synthetic guanylhydrazones. Comparing the empirical approach employed in the HPLC method development to the DoE approach employed in the UHPLC method development, we can conclude that the factorial design made the method development faster, more practical and rational.

## Figures and Tables

**Table 1:** Validation results of HPLC-DAD and UHPLC-DAD method for determination of guanylhydrazone derivatives.

Chromatographic Method	Validation Parameters		LQM010		LQM014		LQM017	
	Linearity (n = 3)		*r^2^*: 0.9995 *y* = 42.04453*x* + 6.54327		*r^2^*: 0.9999 *y* = 74.20108*x* + 0.66868		*r^2^*: 0.9994 *y* = 62.60385*x* + 0.43507	
	Specificity, SI		979		973		959	
			%		%		%	
	Accuracy (n = 5), µg·mL^−1^	8	99.71 ± 1.67		98.69 ± 1.85		100.22 ± 1.86	
		10	100.46 ± 1.34		101.47 ± 0.24		99.71 ± 1.36	
		12	99.49 ± 1.79		98.71 ± 1.50		100.15 ± 1.25	
HPLC			Mean Area ± RSD		Mean Area ± RSD		Mean Area ± RSD	
	Precision (n = 6), 10 µg·mL^−1^	Intra-day	58046 ± 1.48		101134 ± 2.00		79412 ± 1.24	
		Inter-day	56976 ± 2.81		101459 ± 1.56		78202 ± 2.20	
			Mean Area ± RSD	RT	Area	RT	Area	RT
	Robustness Flow (mL·min^−1^)	1.50 ± 0.05	556.53 ± 2.07	5.08	1019.33 ± 2.34	2.64	765.33 ± 2.54	2.18
			%	RT	%	RT	%	RT
	Robustness pH	3.50 ± 0.05	561.04 ± 1.76	5.10	1027.50 ± 1.64	2.60	772.81 ± 1.61	2.20
	Linearity (n = 3)		*r^2^*: 0.9994 *y* = 5092.94*x* − 180.59		*r^2^*: 0.9997 *y* = 8464.98*x* − 533.43		*r^2^*: 0.9997 *y*= 8215.21*x* − 522.16	
	Specificity, SI		999		999		1000	
			%		%		%	
	Accuracy (n = 5), µg·mL^−1^	8	101.62 ± 1.92		99.12 ± 1.35		100.48 ± 1.42	
		10	99.32 ± 0.24		99.07 ± 0.97		99.48 ± 1.34	
		12	100.23 ± 1.45		100.30 ± 0.76		100.33 ± 1.33	
UHPLC			Mean Area ± RSD		Mean Area ± RSD		Mean Area ± RSD	
	Precision (n = 6), 10 µg·mL^−1^	Intra-day	50,277 ± 0.53		80,625 ± 0.84		80,742 ±1.27	
		Inter-day	50,134 ± 0.43		80,321 ± 0.68		80,894 ± 0.63	
			Area	RT	Area	RT	Area	RT
	Robustness Flow (mL·min^−1^)	0.50 ± 0.05	46,917.61 ± 1.66	3.40	76,600.00 ± 1.29	1.67	75,945.22 ± 1.63	1.40
			Area	RT	Area	RT	Area	RT
	Robustness pH	3.50 ± 0.05	46,709.83 ± 1.93	3.40	76,835.67 ± 1.05	1.67	75,542.72 ± 1.53	1.40

*r^2^* = regression analysis of curve; SI = similarity index; % = recovery percentage mean ± relative standard deviation; RSD = relative standard deviation; RT = retention time in minutes.

**Table 2:** Conditions design of experiments (DoE) approach to development of an UHPLC method.

Factor	Levels		
	−1	0	+1
Column length, mm	30	50	75
Flow rate, mL min^−1^	0.3	0.4	0.5
Mobile phase composition, MeOH:H_2_O	60:40	65:35	70:30
